# Research highlights for issue 4: applied evolution in fisheries science

**DOI:** 10.1111/eva.12262

**Published:** 2015-04-21

**Affiliations:** Evolutionary Applications

The pressure on both natural and managed fish stocks to keep pace with worldwide consumption presents a number of critical challenges, including the prevention of population collapse, management of disease, and understanding of the impact that fishing practices may have on life history traits. Addressing such challenges requires the integration of data from long term population monitoring, empirical work, theoretical analysis, and implementation of policy change. Fortunately, many fish populations have been monitored either actively or passively over long periods of time, generating some of the best datasets with which to characterize the impact of human-mediated selection on population-level change.

The intensity of selection acting on fished populations has long been predicted to significantly impact upon life history traits. In a recent theoretical exploration of the consequences of commercial fishing, Lise Marty and coauthors highlight how exploitation of fish populations can lead to slower growth, early maturation, and higher investment in reproduction within stocks (Marty et al. 2015). The authors use an individual-based eco-genetic model to examine harvest-induced genetic change and show not only that fishing can influence life history trait evolution, but also that it can reduce effective population size and erode additive genetic variation. Together, they argue, these effects are likely to hinder recovery even after intense fishing has ceased.

Another recent theoretical analysis examining the consequences of fisheries on stock populations suggests that common fishing policies can result in disruptive selection for maturation strategies (Landi et al. 2015). Using an eco-evolutionary model, Pietro Landi and colleagues demonstrate how the interplay between adaptation of fish stocks and adaptation of fisheries policy can lead to dimorphism within populations, with some individuals reaching maturation early and others late, investing instead in growth and fecundity. This work highlights the potentially complex outcomes of size-selective harvesting and the need for adaptive policies that take into account evolutionary change of fish populations.

Harvest-mediated shifts in life history have thus far been demonstrated under a variety of scenarios. Recent empirical work examining size and weight distributions of exploited sea cucumber populations in Turkey finds evidence for the loss of larger size classes, as predicted from intensive size-dependent harvesting (González-Wangüemert et al. 2015). By comparing fishery and nonfishery populations, Mercedes González-Wangüemert and collaborators show that individuals from protected populations tend to be larger and heavier, with higher genetic diversity than those from exploited populations. Given that sea cucumber over-exploitation is a relatively recent and growing phenomenon, this work offers an important new data point in a rapidly expanding body of evidence for rapid fisheries-mediated evolutionary change in fish stocks.

Finally, just like natural populations, managed fish stocks face a constant onslaught of pests and pathogens. This is further exacerbated by high population densities, increased movement of disease agents among populations, and potentially by selection for desirable traits that are negatively correlated with resistance. A recent review by Kevin Lafferty and coauthors examines the ongoing challenges associated with controlling the emergence and spread of disease within fisheries and aquaculture, highlighting a number of significant infectious agents with severe economic impacts. The authors further explore how the novel evolutionary environment of fish farms might influence pathogen evolution, for example leading to higher virulence, and whether host resistance is likely to evolve under current fishing practices (Lafferty et al. 2015).

For bacterial pathogens within fish farms, there has been increasing interest in the use of bacteriophages as control agents. Although there is still uncertainty in regard to best practice for the application of phages within these complex environments, work from the laboratory suggests this as a promising avenue, especially in combination with other control measures. Recent work by Daniel Castillo and collaborators undertook a study on the common fish pathogen, *Flavobacterium psychrophilum*, to examine both the genetic changes underlying the evolution of bacterial resistance to phage and the physiological changes associated with such resistance (Castillo et al. 2015). They found numerous mutational changes underlying resistance, suggesting that resistance can be attained relatively easily and via a number of mechanisms, but also that these resistance mutations are often associated with a loss of virulence when measured *in vitro*.

Overall, the application of evolutionary and ecological theory to fisheries management over the last few decades has proven invaluable, but there remains a great need for further empirical and observational datasets testing the predictions put forward. Furthermore, translating such knowledge into policy change continues to present a formidable challenge for the field.
